# Global burden of disease for colorectal cancer due to diet low in whole grains from 1990 to 2021 and its projections to 2050: analysis of the global burden of disease study 2021

**DOI:** 10.3389/fnut.2025.1592425

**Published:** 2025-08-07

**Authors:** Mei Yang, Gansheng Huang, Feng Jiang, Zaijin Guo

**Affiliations:** The Eighth Hospital of Wuhan, Wuhan, China

**Keywords:** global burden of disease, colorectal cancer, epidemiological, cancer, diet low in whole grains

## Abstract

**Introduction:**

Colorectal cancer is a highly prevalent and significantly lethal digestive malignancy worldwide. This study aims to reveal the evolution of the disease burden of CRC associated with a low-grain diet from 1990 to 2021, to predict future trends, and to provide a scientific basis for differentiated prevention and control strategies.

**Methods:**

The study integrated GBD 2021 data covering 204 countries and territories to assess the disease burden of CRC associated with a low-grain diet by the number of deaths, disability-adjusted life-years (DALYs), age-standardized mortality rates (ASMR), and Age-standardized DALYs rates (ASDR). Trends were quantified using Estimated Annual Percentage Change (EAPC) and disease burden was projected to 2050 using Bayesian Age-Period-Cohort (BAPC) modeling. Decomposition analyses explored the impact of population growth, aging, and epidemiologic changes on burden.

**Results:**

Between 1990 and 2021, the number of CRC deaths associated with a low proportion of whole grain diets worldwide increased by 82.94%, and disability adjusted life years increased by 70.3%. There is significant heterogeneity between regions: regions with high Social Population Index (SDI) have the highest decrease rate, while regions with low to medium SDI have the fastest increase rate. Age analysis shows that the disease burden is highest in the 50–85 age group, with males having a significantly higher risk than females. The BAPC model predicts that by 2050, global ASMR and ASDR will further decline, and decomposition analysis shows that population growth is the main reason for the increase in burden.

**Conclusion:**

Age-standardized mortality rates and ASDR for CRC due to low whole grain diets declined globally between 1990 and 2021, but the absolute number of cases continues to increase. Low whole grain diet is an important modifiable factor in the disease burden of CRC, with significant age, sex, and regional heterogeneity in its impact. Bayesian BAPC model predictions showed a decreasing trend in ASMR and ASDR for colorectal cancer disease burden associated with low grain diets, but the absolute burden continued to increase due to increased aging. Decomposition analyses showed that population growth was the main cause of the increasing burden.

## 1 Background

Colorectal cancer is a common and lethal digestive system malignancy posing significant public health threats globally. In addition, the survival of patients with CRC is strongly dependent on the stage of cancer at diagnosis. The 5-years relative survival rate for the localized stage is higher than 90%, whereas that for the distant stage is less than 10% (1, 2), while incidence rates in high-income countries have stabilized or declined in some regions, they exhibit marked upward trends in low- and middle-income countries (3). According to the 2022 Global Cancer Statistics, CRC accounted for over 1.9 million new cases and 935,000 deaths worldwide. The age distribution of its disease burden exhibits a “bimodal” pattern: younger adults (15–59 years) demonstrate accelerated incidence rates, while older adults (≥ 60 years) remain the primary population burdened by this disease (4). The etiology of CRC is highly complex, involving interactions between genetic mutations, environmental factors, dietary patterns, and gut microbiota alterations (5–7).

The etiology of colorectal cancer (CRC) is predominantly attributed to profound environmental and lifestyle changes in populations. Extensive research has identified multiple risk factors for CRC, including obesity, smoking, alcohol consumption, red meat and processed meat intake, dietary patterns characterized by high sugar content, low calcium and vitamin D levels, physical inactivity, and chronic diseases such as diabetes and inflammatory bowel disease (7–10). Recent studies have emphasized that metabolic inflammation is a key pathway in the development of CRC due to a low whole-grain diet, and that the process involves dysbiosis of the intestinal flora, which promotes the release of pro-inflammatory factors and impairs intestinal barrier function (11). Dietary factors have long been recognized as significant contributors to carcinogenesis (12). Epidemiological studies consistently demonstrate that reduced consumption of dairy products, dietary fiber, and calcium supplements may increase CRC risk (13), with particular emphasis on the strong association between excessive red meat and processed meat consumption and CRC incidence (14, 15). As a major modifiable risk factor, dietary patterns have received increasing research attention in recent years, particularly highlighting the emerging health implications of Diet low in whole grains (16).

Whole grains (e.g., brown rice, oats, whole wheat) are rich in dietary fiber, antioxidants, and phytochemicals, with insufficient intake strongly associated with increased colorectal cancer (CRC) risk (17, 18). The World Health Organization (WHO) recommends a daily intake of at least 50 grams of whole grains, and the Dietary Guidelines for Americans (2020–2025) recommend consuming 6 ounce equivalents of grains per day, with at least half of that coming from whole grain sources (19). However, epidemiological evidence reveals a paradoxical trend where economic development correlates with rising refined grain consumption and declining whole grain intake - particularly pronounced in rapidly urbanizing societies afflicted by fast-food culture proliferation (20, 21). Diet low in whole grains contribute to CRC pathogenesis through multiple interconnected mechanisms: reduced dietary fiber impairs intestinal motility, extending carcinogen exposure time; gut microbiota dysbiosis induces chronic inflammation; insulin resistance and oxidative stress collectively promote epithelial cell hyperproliferation (22). Notably, existing research remains disproportionately focused on red meat carcinogenicity, demonstrating critical gaps in systematic evaluations of Diet low in whole grainsary patterns, age-stratified analyses (e.g., distinct young adult versus elderly population vulnerabilities), and geosocioeconomic disparities in dietary behaviors. This research deficit highlights the urgent need for multidimensional investigations addressing these understudied dimensions.

The Global Burden of Disease (GBD) study, initiated in 1990, aims to systematically evaluate health outcomes for diverse diseases through timely, relevant, and evidence-based assessments. The latest GBD data released in May 2024 covers 2021 measurements across 204 countries and regions, incorporating 288 causes of death, 371 diseases and injuries, and 88 risk factors. This comprehensive framework quantifies incidence rates, mortality rates, disability-adjusted life-years (DALYs), and other critical health metrics (23). A number of macroscopic studies on the association of nutritional intake with colorectal cancer burden, such as high red meat diets and low calcium intake diets, have been provided in previous studies (24–27). Accelerated population aging and diversifying dietary patterns underscore the urgency of predicting future disease burden trajectories for informed public health policymaking. This study therefore integrates GBD 2021 data to analyze temporal patterns (1990–2021) of Diet low in whole grains-related CRC burden using mortality and DALYs as primary indicators. Beyond descriptive analysis, it explores underlying drivers of evolving disease burden dynamics, including demographic shifts (population growth and aging), epidemiological transitions, and dietary behavioral changes. These findings offer actionable evidence for targeted interventions and resource allocation strategies to address CRC’s escalating global burden effectively.

Our objective was to investigate the global, regional, and national burden of CRC deaths and disability-adjusted life years due to low whole grain diets between 1990 and 2021. Trends in burden to 2050 were also projected, providing a scientific basis for differentiated prevention and control policies for specific populations and regions.

## 2 Materials and methods

### 2.1 Data sources and definitions

Data were extracted from the Global Health Data Exchange website for the period 1990–2021,^1^ stratified by gender, age, and region. These datasets encompass colorectal cancer (CRC)-specific mortality, disability-adjusted life-years (DALYs), age-standardized mortality rates (ASMR), and age-standardized DALY rates (ASDR). Using this comprehensive dataset, we extensively analyzed epidemiological trends of Diet low in whole grains CRC burden across genders, age groups, and regions. Simultaneously, we investigated the relationship between the Social Demographic Index (SDI) – a composite index integrating per capita income, average educational attainment, and fertility rate – and disease burden patterns. The SDI framework categorizes 204 countries and regions into five quintiles: Low SDI (< 0.45), Medium SDI (≥ 0.45 and < 0.61), Upper-Medium SDI (≥ 0.61 and < 0.69), High-Medium SDI (≥ 0.69 and < 0.80), and High SDI (≥ 0.80). This methodologically rigorous analysis enables characterization of spatial-temporal variations in CRC burden attributable to dietary patterns, while simultaneously illuminating how sociodemographic development influences disease prevalence across demographic strata and geographic regions (28).

In the 2021 GBD study, the burden associated with dietary risk was estimated using a comparative risk assessment framework. Input data sources were mainly from nutritional surveys, such as the 24-h dietary recall, the food frequency questionnaire and the Food and Agriculture Organization of the United Nations (FAO) Dietary Risk Assessment Framework (DRAF). Whole grain intake is defined as the average daily consumption (in grams/day) of whole grains and their products, including breakfast cereals, breads, rice, cookies, muffins, tortillas, pancakes, pasta and other related sources. A low whole grain diet is defined as an average daily intake of less than 140–160 g/day (24, 29).

### 2.2 Statistical analysis

The Estimated Annual Percentage Change (EAPC) quantifies the average yearly percentage change in a health indicator over a specified time period. It is derived from a linear regression model that assumes a linear relationship between the natural logarithm of the Age-Standardized Rate (ASR) and the calendar year. This relationship is expressed as: ln(ASRt) = α + βt + σ, where α is the intercept, β was the slope (i.e., the regression coefficient), and σ was the error term (30). The 95% confidence interval (CI) is derived from the linear regression model.

Estimated Annual Percentage Change was employed to assess the rate of change for each indicator across 204 countries and regions worldwide from 1990 to 2021. The calculation formula is:


EAPC=[exp⁢(β)-1]×100%

We analyzed colorectal cancer (CRC) burden among adults aged ≥ 25 years at the national level globally. To account for demographic variations, we employed age-standardized mortality rates (ASMR) and disability-adjusted life-year (DALY) rates as indicators to better reflect true incidence and mortality patterns, with all rates expressed per 100,000 population. Additionally, we applied the estimated annual percentage change (EAPC) to evaluate temporal trends in ASMR and ASDR. Statistical significance was determined through 95% confidence intervals (CI): EAPC values > 0 indicated increasing annual trends, while EAPC < 0 denoted decreasing trends. An EAPC close to 0 suggested stable disease burden over time. This analytical framework enables robust characterization of CRC burden dynamics across different populations while accounting for both temporal and spatial heterogeneity (31).

### 2.3 Decomposition analysis

Decomposition analysis in epidemiology dissects changes in health indicators to reveal the contributions of population growth, aging populations, risk factor prevalence, or medical advancements (32). Our assessment quantified the relative impacts of these factors, including demographic expansion, population aging trajectories, and epidemiological transitions.

### 2.4 Age-period-cohort model

The age-period-cohort (APC) model integrates age, period, and cohort effects to analyze and predict disease trends while accounting for demographic dynamics (33). The model provides a nuanced approach to predicting disease trends by taking into account the complex interactions of population changes over time (34). All statistical analyses and visualizations were conducted using R statistical software (Version 4.2.3), with statistical significance defined as two-tailed *p* < 0.05.

### 2.5 Bayesian age-period-cohort modeling

Using GBD data from 1990 to 2021, we projected colorectal cancer (CRC) burden for 2022–2050. Our methodology comprised two principal steps: First, we compiled global and regional age-specific (5-years intervals) mortality and disability-adjusted life-years (DALY) rates attributable to Diet low in whole grains from 1990 to 2021. Subsequently, we employed a Bayesian age-period-cohort (BAPC) model to forecast future disease burden. This Bayesian framework accounts for individual age groups, event timing (periodic effects), and birth cohort memberships through probabilistic inference (35). Bayesian APC models demonstrate particular advantages in cancer burden projection by avoiding parametric assumptions about temporal trends, a strength supported by their widespread adoption in epidemiological research (36, 37). Bray’s comparative analysis demonstrated that Bayesian APC models produce more plausible predictions compared to classical APC approaches when evaluating linear power-law models (38). All statistical computations and visualizations were performed using R software (Version 4.2.3), primarily uses the “GlobalBurdenR” package (33).

## 3 Results

### 3.1 Global burden of colorectal cancer attributable to diet low in whole grains (1990–2021)

Globally, the number of deaths from CRC due to Diet low in whole grains shows a significant upward trend from 1990 to 2021, rising from 101,812 (95% CI: 42588.20–151170.08) in 1990 to 186,625 (95% CI: 76,126.73–284,803.37) in 2021, representing an increase of about 82.94% in the number of deaths. Despite the increase in absolute numbers, the Age Standardized Mortality Rate (ASMR) has shown a slight downward trend from 2.79 per 100,000 in 1990 to 2.21 per 100,000 in 2021, a decrease of 20.79% (Supplementary Table 1).

Globally, the DALYS for CRC due to Diet low in whole grains showed a significant upward trend from 1990 to 2021, increasing from 2540867.41 (95% CI: 1050794.36–3754415.59) in 1990 to 4327218.86 (95% CI: 1754865.24–6578232.30) in 2021. (1) The DALYS for CRC due to Diet low in whole grains showed a significant upward trend from 1990 to 2021, increasing from 2540867.41 (95% CI: 1050794–36,3754415.59) in 1990 to 4327218.86 (95% CI: 1754865.24–6578232.30) in 2021. As with the age-standardized mortality rate (ASMR), the ASDR decreased from 63.47/100,000 (95% CI: 26.35–93.84) in 1990 to 50.19/100,000 (95% CI: 20.37–76.30) in 2021 (Supplementary Table 2).

### 3.2 Regional burden of colorectal cancer due to diet low in whole grains, 1990–2021

The highest number of deaths and age-standardized death rates among the five SDI regions were in High SDI 60473.36 (95% CI: 25199.86–92184.17), High-middle SDI 2.89 (95% CI: 1.17–4.38), and the lowest were in Low-middle SDI 15442.23 (95% CI: 6372.49–22973.55), 1.11 (95% CI: 0.46–1.65). In terms of temporal trends, ASMR was aggravating in Low-middle SDI (EAPC = 0.45) and decreasing in the rest of the area (Supplementary Table 1).

The highest DLAYs, ASDR were in Low-middle SDI 430073.38 (95% CI: 176445.15–643409.64), High-middle SDI 66.60 (95% CI: 26.65–100.77) and the lowest were in Low-middle SDI 28.05 (95% CI: 11.53, 41.95), High SDI 1205653.28 (95% CI: 500387.21–1815640.18), respectively. The time trend showed that ASDR was increasing in Low-middle SDI (EAPC = 0.36) and decreasing in the rest of the area, which was consistent with the trend of the mortality indicator (Supplementary Table 2).

The highest number of deaths and age standardized death rate among the 21 GBD regions were East Asia 52,282.82 (95% CI: 20,975.52–83,298.33), Central Europe 4.13 (95% CI: 1.71–6.20). In terms of temporal trend, Southern Sub-Saharan Africa region showed the largest increase in ASMR of 1.00, while the remaining 10 regions showed an increasing trend in ASMR (EAPC > 0) and 11 regions showed a decreasing trend (EAPC < 0) (Supplementary Table 1).

The highest DLAYs and ASDR were found in East Asia 1295744.16 (95% CI: 524659.79–2051943.02), Central Europe 127.75 (95% CI: 52.80–192.67) (Supplementary Table 1). In terms of temporal trends, Southern Sub-Saharan Africa region has the largest upward trend in ASDR at 1.10, while the remaining 10 regions show an increasing trend in ASDR (EAPC > 0) and 11 regions show a decreasing trend in ASDR (EAPC < 0) (Supplementary Table 2).

### 3.3 National burden of colorectal cancer due to diet low in whole grains, 1990–2021

**FIGURE 1:**
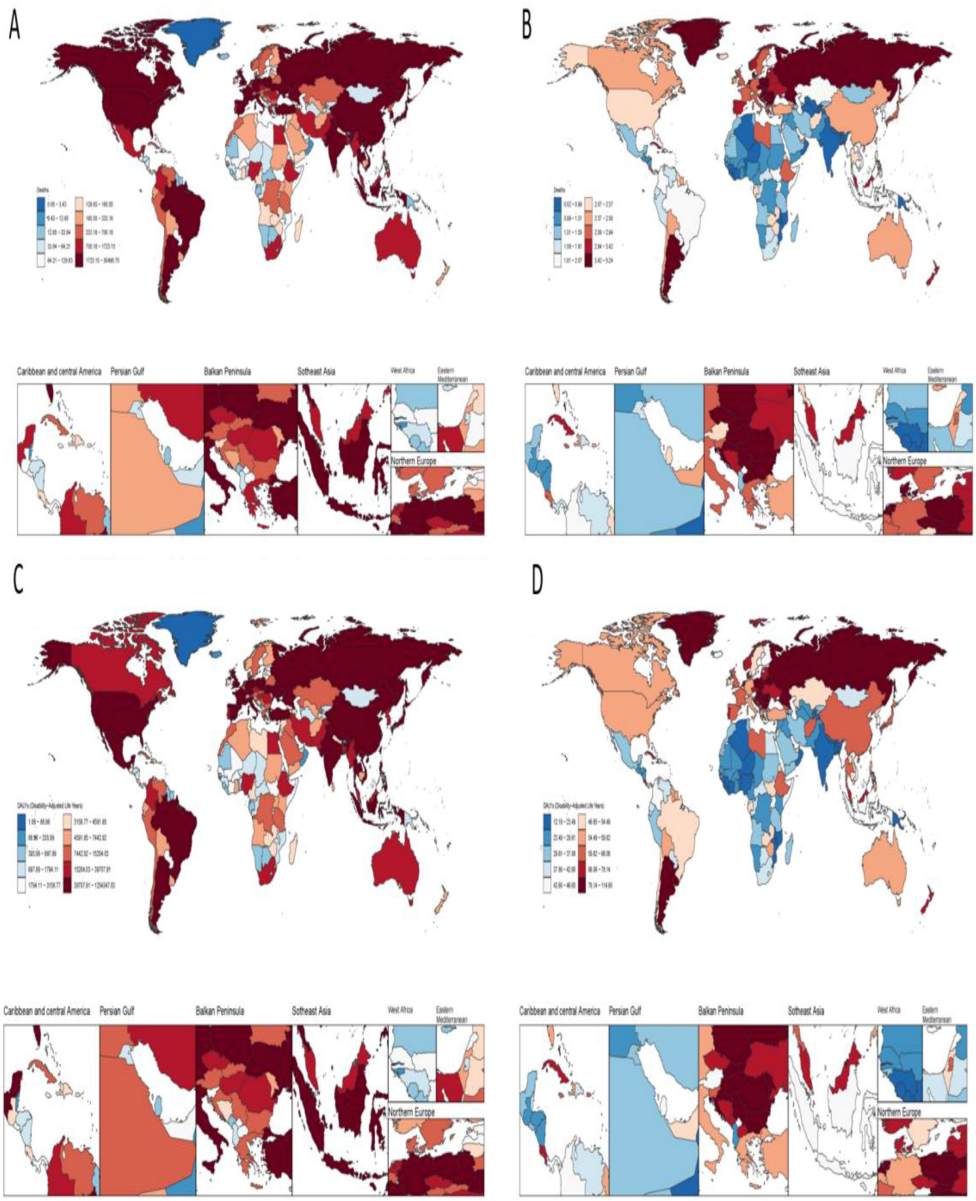
Disease burden of colorectal cancer due to low whole grain intake in 204 countries and territories. **(A)** Deaths **(B)** ASMR **(C)** DALYs **(D)** ASDR.

**FIGURE 2:**
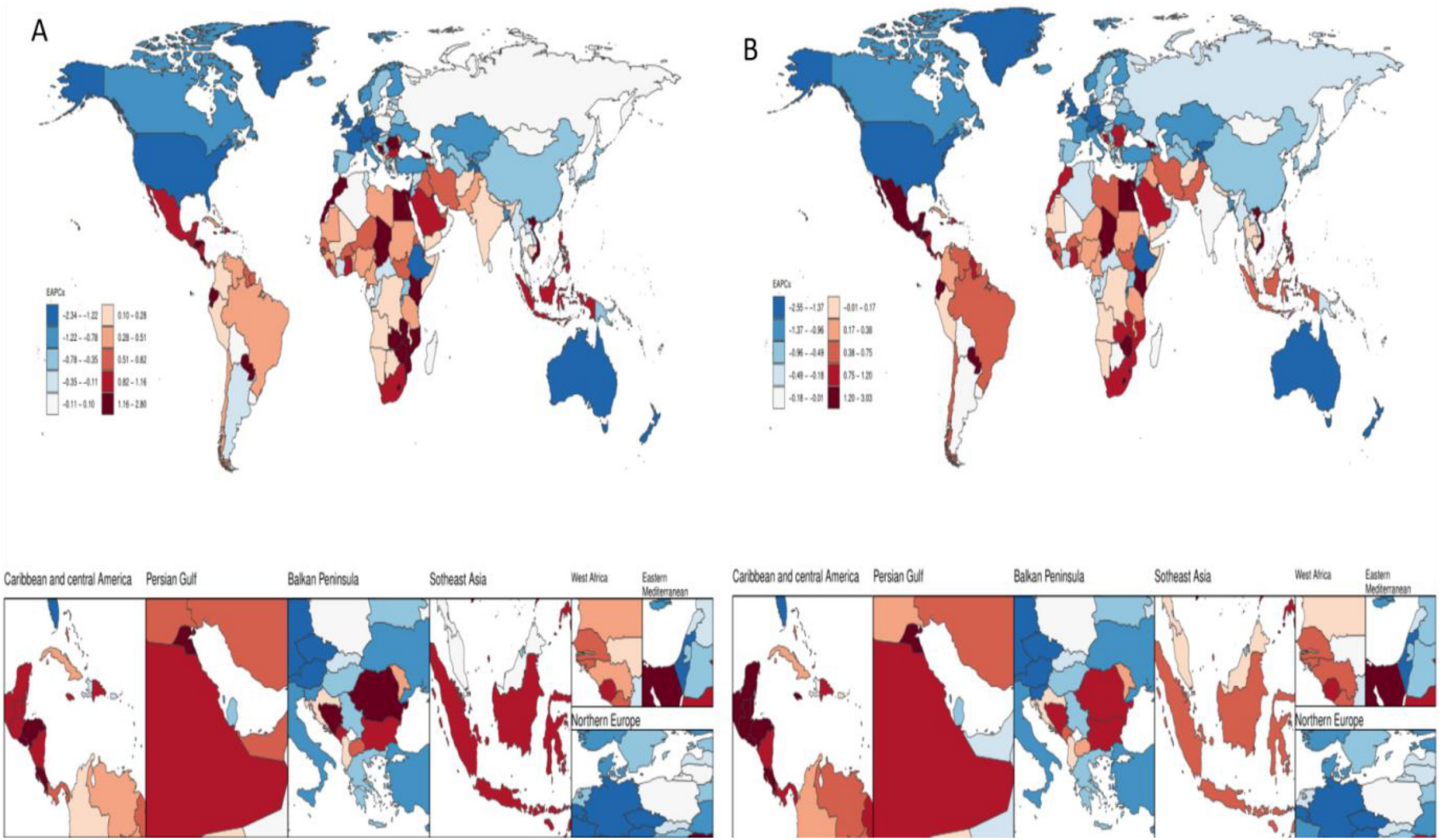
Temporal trends in the burden of disease for colorectal cancer due to low whole grain intake in 204 countries and regions. **(A)** EAPCs for ASMR **(B)** EAPCs for ASDR.

In 2021, China had the highest number of colorectal cancer deaths due to Diet low in whole grains at 49,990.79 (95% CI: 20,099.58–79,928.55) Eastern Republic of Uruguay had the highest ASMR due to colorectal cancer due to Diet low in whole grains at 5.18% (95% CI: 2.09–7.72) The analysis of longitudinal temporal trends showed that. DALYs showed an increasing trend in 112 out of 205 countries (EAPC > 0), and 1 country Kingdom of Thailand remained unchanged (EAPC = 0) of which the top three increasing trends were Kingdom of Lesotho (EAPC = 2.77), Republic of Cabo Verde (EAPC = 2.44), and Arab Republic of Egypt (EAPC = 2.12). The highest DALYs were in China at 1241927.75 (95% CI: 503164.75, −1978508.32) and the highest ASDR was in Hungary at 113.75 (95% CI: 47.61–173.36). Longitudinal time trend analysis showed that 99 out of 204 countries showed an increase in DALYs, with the top three being Kingdom of Lesotho (EAPC = 3), Republic of Cabo Verde (EAPC = 2.11), Republic of Costa Rica (EAPC = 1.99) See Figures 1A–D, 2A, B for details.

### 3.4 Age-sex-time association analysis of colorectal cancer due to diet low in whole grains, 1990–2021

**FIGURE 3:**
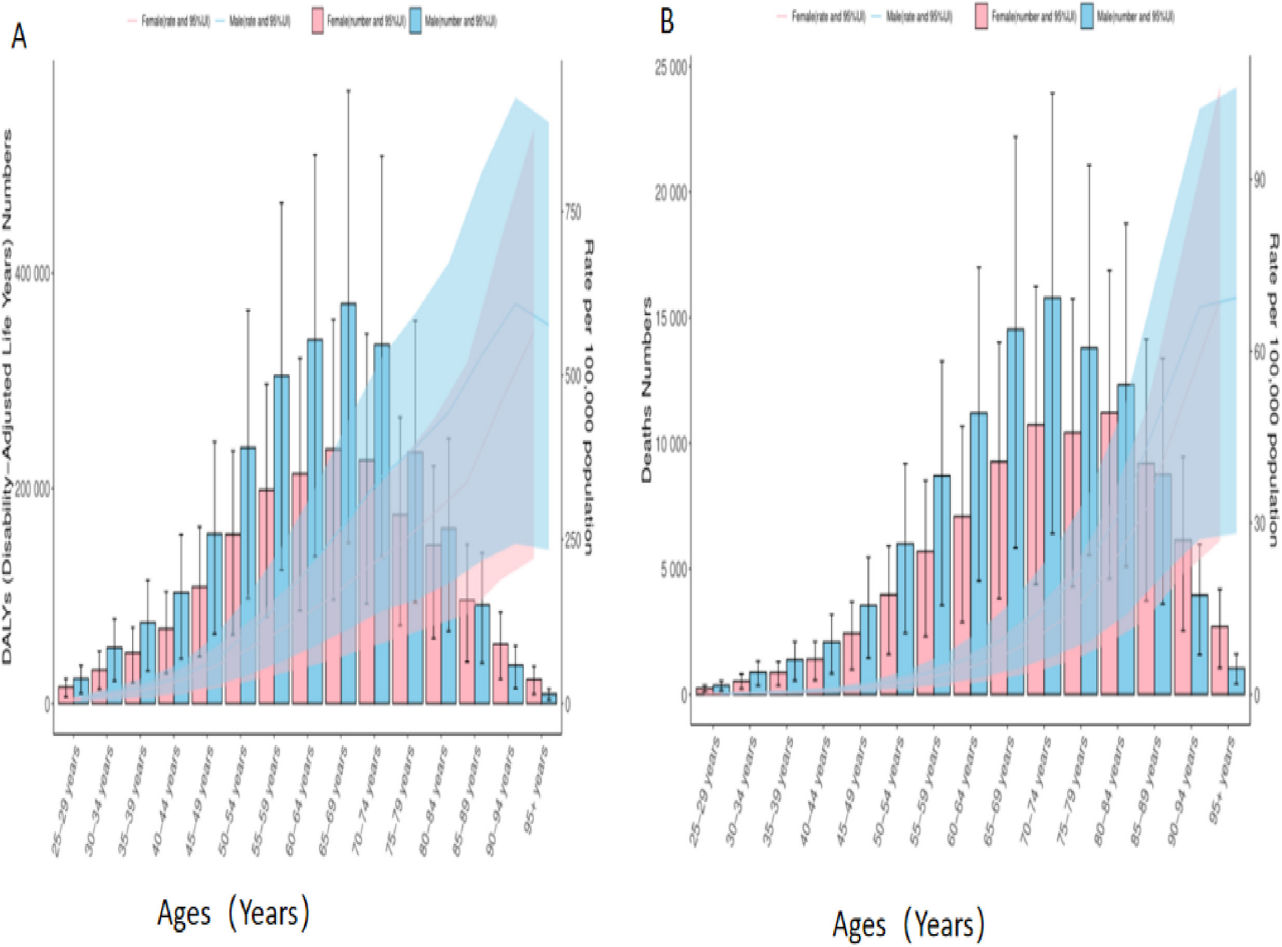
Age-sex biaxial coordinate plot of colorectal cancer disease burden due to Diet low in whole grains. **(A)** DALYs **(B)** Deaths.

Age-sex association analyses showed that in mid-2021, the DALYs, number of deaths from colorectal cancer were significantly higher and showed a substantial increasing trend in individuals aged 50–85 years. 25–44 years were comparatively low. The DALYs and number of deaths from colorectal cancer increased with age from 25 to 69 years, but decreased with age at 70 years and beyond. Gender analysis showed that the burden of disease was greater for males than for females in all different age groups, as shown in Figure 3.

### 3.5 Association of disease burden of colorectal cancer due to diet low in whole grains with SDI, 1990 to 2021

**FIGURE 4:**
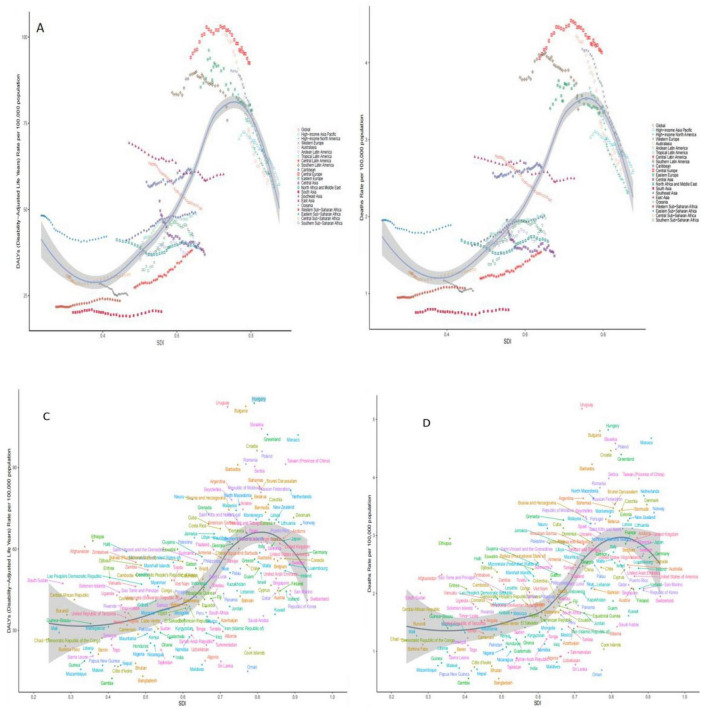
Trends in burden of disease with SDI for 21 GBD regions and 204 countries and territories with colorectal cancer due to Diet low in whole grains globally, 1990–2021. **(A)** ASDR for 21 regions **(B)** ASMR for 21 regions **(C)** ASDR for 204 countries or regions **(D)** ASMR for 204 countries or regions.

In this study, data from 21 different geographic super-regions were analyzed, and a non-linear relationship was observed between SDI and ASDR and ASMR. Specifically, the burden of disease for colorectal cancer due to Diet low in whole grains showed a slow decreasing trend with increasing SDI, with the lowest burden of disease at around 0.4, followed by a slow increasing trend with increasing SDI. The disease burden was highest at around 0.75, after which it was decreasing with increasing SDI. The burden of disease for Hungary is the fastest rising among the 204 countries, as shown in Figures 4A–D.

### 3.6 Results of the age-period-cohort analysis

**FIGURE 5:**
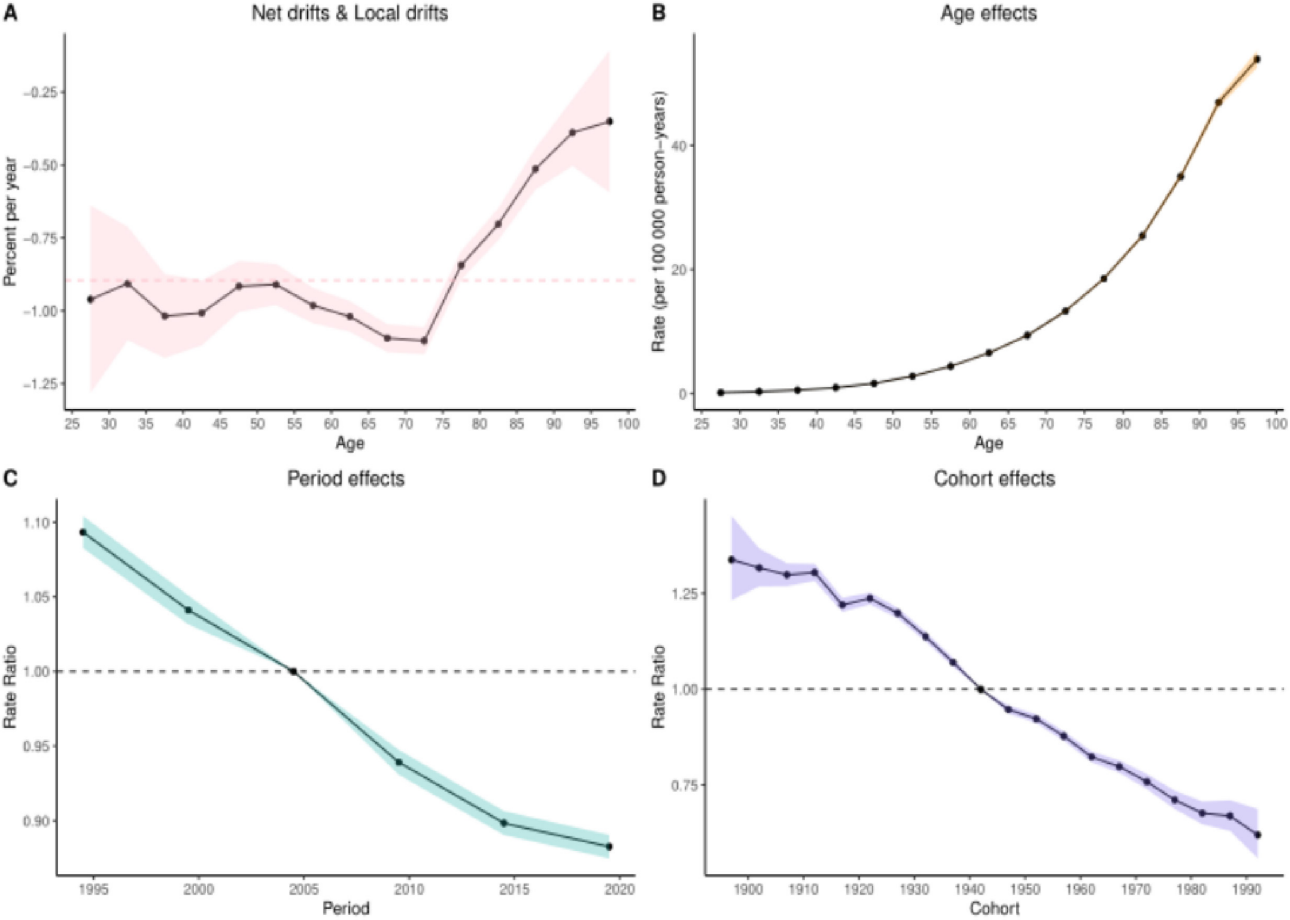
Results of age-period-cohort analyses of mortality. **(A)** Net and localized drift **(B)** Age effect **(C)** Period effect **(D)** Cohort effect.

**FIGURE 6:**
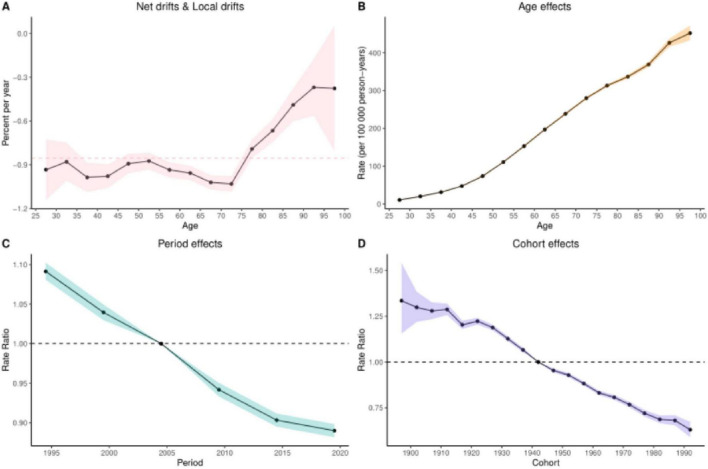
Results of age-period-cohort analysis of DALYs. **(A)** Net and local drift **(B)** Age effect **(C)** Period effect **(D)** Cohort effect.

The age-period-cohort analysis of colorectal cancer (CRC) burden attributable to Diet low in whole grains revealed analogous temporal patterns across mortality and DALYs. When examining age effects for these two burden metrics, the net drift and local drift (Panel A) further validated dynamic age-related risk variations. This section quantifies both net drift (overall temporal trends independent of age structure) and local drift (age-specific annual percentage changes in DALY rates across different age groups), See Figures 5, 6 illustrating how the magnitude of DALY rate changes evolves with advancing age. The age effect analysis (Panel B) demonstrated that both DALYs and mortality rates increased progressively with age after controlling for period and cohort influences, reflecting inherent biological aging mechanisms. Conversely, the period effect analysis (Panel C) revealed a significant downward trend in disease burden between 1995 and 2021, suggesting secular improvements in social, environmental, or medical conditions associated with these decades. Finally, the cohort effect analysis (Panel D) highlighted long-term birth cohort influences, demonstrating that later-born cohorts (born in more recent decades) exhibited substantially lower CRC burden compared to earlier generations–a pattern consistent with socioeconomic advancements. Quantitative details supporting these analyses are provided in Supplementary Tables 3, 4.

### 3.7 Predicting the disease burden of colorectal cancer due to diet low in whole grains using bayesian age-period-cohort modeling

**FIGURE 7:**
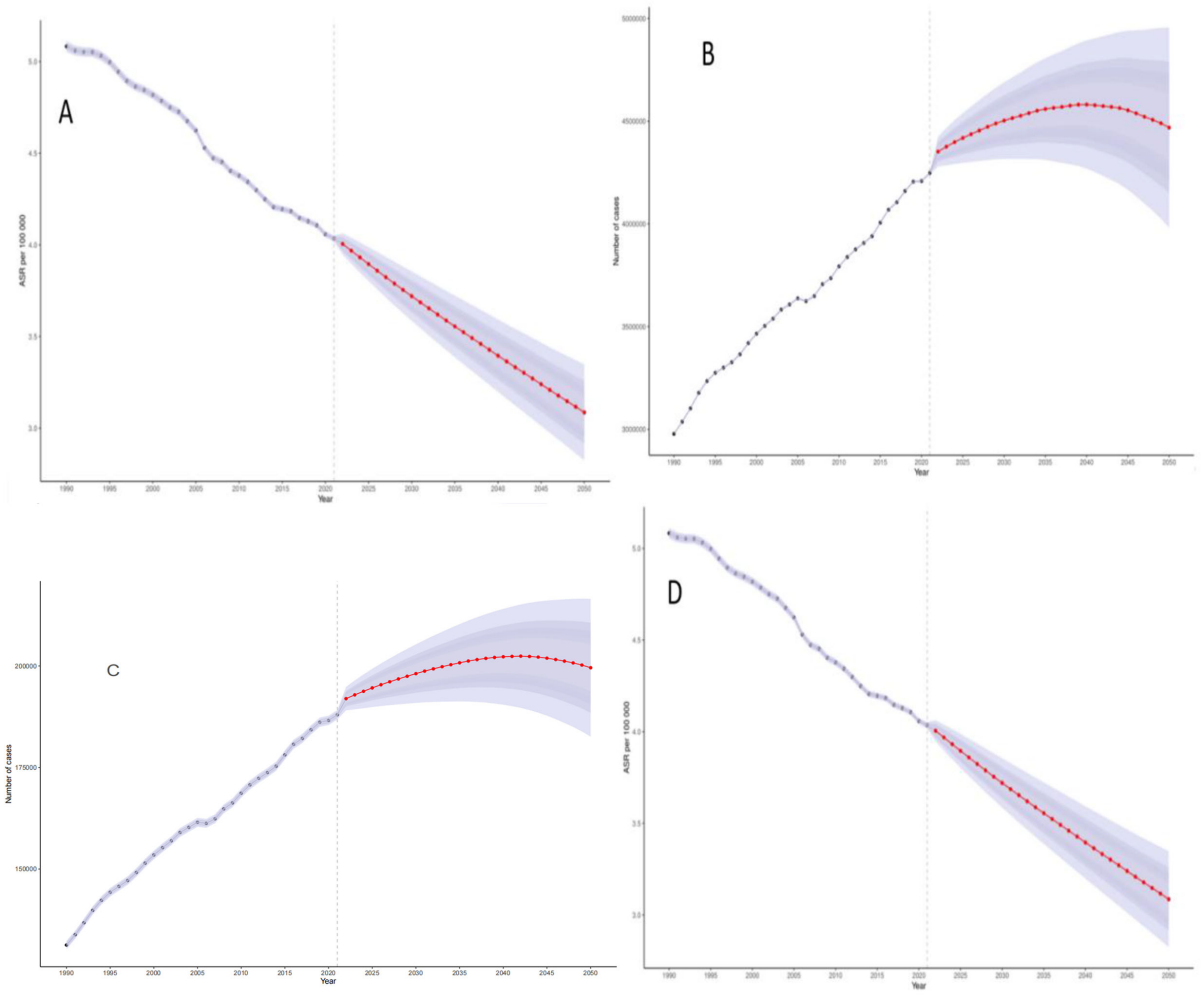
Bayesian Age-Period-Cohort (BAPC) model projections of disease burden of colorectal cancer due to Diet low in whole grains through 2050. **(A)** ASDR **(B)** DALYs **(C)** Deaths **(D)** ASMR.

We examined the change in disease burden of colorectal cancer due to Diet low in whole grains globally over time from 1990 to 2021, and the predictive analysis showed that the relative indicators of colorectal cancer due to Diet low in whole grains will further decrease but the absolute indicators will increase globally from 2022 to 2050. In 2050, the ASDR, DALYs, Deaths, and ASMR for colorectal cancer due to Diet low in whole grains will decrease to 69.10 (95% CI: 61.56–3.348), 4468600 (95% CI: 3981149–4956051)/100,000 person-years, 199566 (95% CI: 182609–216523)/100,000 people, 3.086 (95% CI: 2.824–3.348)/100,000 people See Figures 7A–D for details. In addition, our analysis of the prediction of different age groups found that the DALYs and number of deaths of different age groups also showed a significant upward trend, and the older the age, the more pronounced the upward trend was, as shown in Supplementary Figures 1, 2 for details.

### 3.8 Results of a decomposition analysis of the burden of colorectal cancer disease due to diet low in whole grains, 1990–2021

**FIGURE 8:**
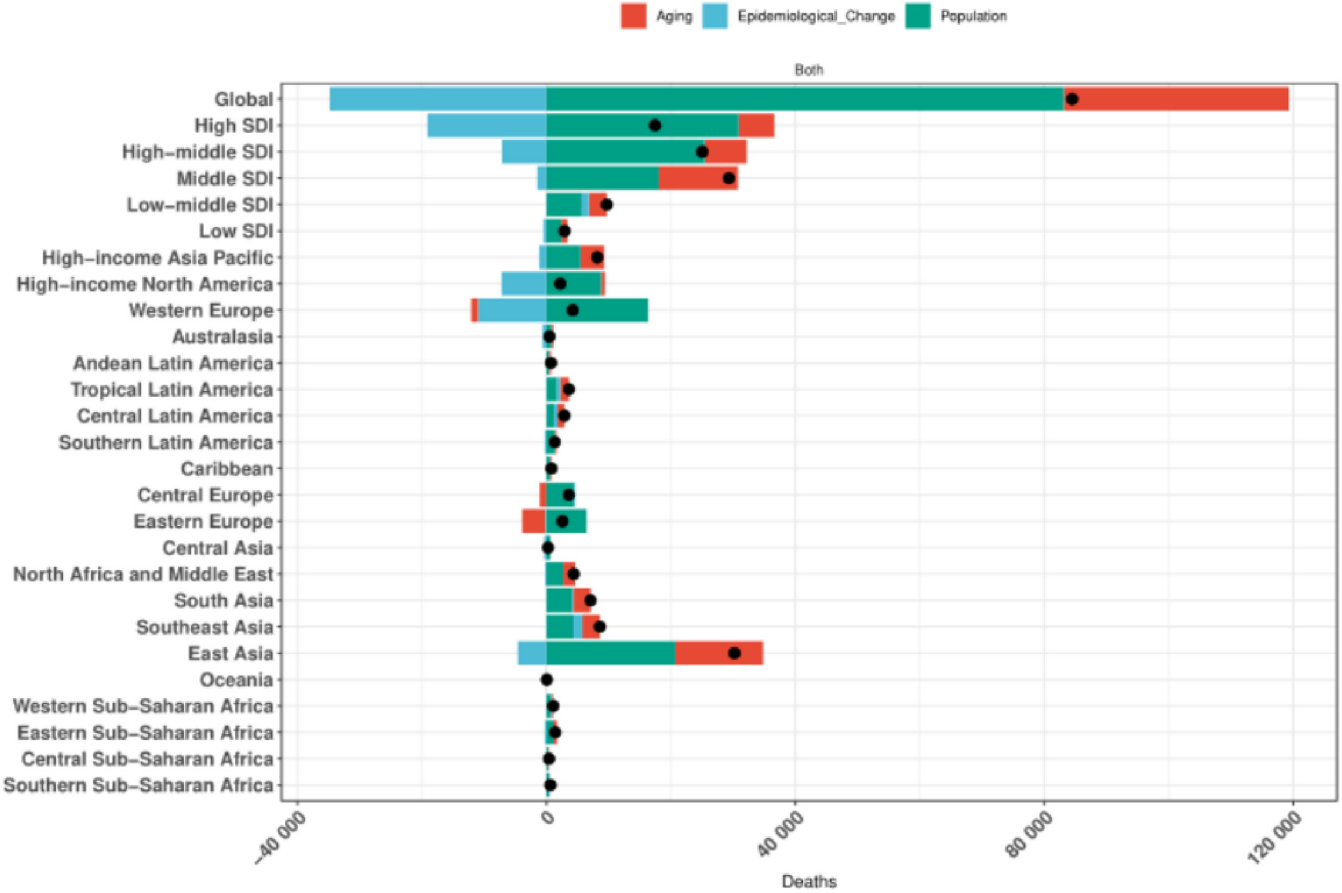
Global and 5 SDIs and 21 regions decomposition analysis results.

The results of the decomposition analyses showed that population growth, aging, and epidemiologic changes in Global, the 5 SDI regions, and the 21 GBD regions exhibited similar patterns of contribution to the impact of colorectal cancer disease burden due to low whole grain intake. Specifically, with the exception of Global, High SDI and High-middle SDI, Western Europe, High-income North America, and Eastern Asia regions, where aging significantly reduced the disease burden, population growth, aging, and epidemiologic changes contributed to the colorectal Population growth was the most significant cause of increased colorectal cancer disease burden due to Diet low in whole grains, as detailed in Figure 8.

## 4 Discussion

Cereals contain a variety of potentially anti-cancer compounds, including antioxidants, trace minerals, phytates, phenolic acids, phytoestrogens, and fiber (39). Common whole grains include wheat, rye, oats, barley, mixed grains, and whole grains plus bran. Many studies on whole grain consumption have found a negative correlation between CRC risk and whole grain intake (40), particularly in individuals aged 70 years and older (8, 41). The mechanistic link between low-grain consumption and CRC risk elevation in elderly populations may involve multiple pathways: dietary fiber deficiency disrupts intestinal motility and carcinogen clearance, chronic inflammatory immune dysregulation promotes epithelial proliferation, and gut microbiota dysbiosis induces procarcinogenic metabolic alterations (42, 43). These physiological changes associated with Diet low in whole grains are amplified in older adults through age-related declines in gut barrier function, immune surveillance, and cumulative molecular damage, collectively promoting tumorigenesis and metastatic progression through genome instability, oncogenic signaling pathway activation, and immunosuppressive tumor microenvironment formation (44–47).

Our global analysis of CRC burden attributable to low-grain intake (1990–2021) revealed divergent temporal patterns: while age-standardized mortality rates (ASMR) and disability-adjusted life-years (DALY) rates (ASDR) decreased by 20.79% and 20.9% respectively, the absolute numbers of CRC deaths and DALYs rose by 82.94% and 70.3% due to population aging and expanding disease prevalence. Notably, a recent study documented that overweight and obese individuals exhibit 18% and 32% higher CRC incidence compared to normal-weight counterparts (48). This aligns with global dietary transitions where excessive high-fat, high-protein consumption–often accompanying low-grain intake–has exacerbated obesity rates, thereby intensifying CRC’s global health burden through synergistic effects on metabolic disorders and colorectal carcinogenesis (49).

Regional heterogeneity in CRC burden reveals significant disparities across SDI quintiles. High-SDI regions (e.g., East Europe, East Asia) exhibit the highest ASMR and ASDR rates, while low/middle-SDI countries (e.g., Lesotho, Cabo Verde) demonstrate the fastest increasing disease burden trajectories (EAPC > 2), underscoring the profound impact of socioeconomic inequalities on health outcomes. Economically advanced regions exhibit high consumption of processed foods and red meat alongside insufficient whole grain intake dietary patterns directly linked to CRC risk elevation. For instance, East Asia’s traditional diet has been increasingly supplanted by high-fat, low-fiber Westernized diets, exacerbating colorectal health challenges (50). Conversely, high-SDI nations are more likely to implement systematic CRC screening programs proven interventions for early detection while rapidly developing regions experience explosive growth in refined grain and processed food consumption, precipitating sharp increases in CRC risk burden.

Low/middle-SDI areas face critical limitations in CRC early-screening infrastructure, leading to frequent late-stage diagnoses, poor treatment outcomes, and accelerated mortality rates (26). Among age and sex differences, the highest burden of disease was found in people aged 50–85 years, with men having a significantly higher risk than women, possibly because physical decline due to advanced age may increase the risk of morbidity and mortality from CRC by promoting the growth of tumor cells and decreasing the ability of the immune system to clear the tumor (51, 52). Age-gender disparities further characterize CRC burden distribution: individuals aged 50–85 years bear the highest disease burden, with males exhibiting significantly elevated incidence, mortality, and DALY rates compared to females (26, 53, 54). This gender gap may stem from both behavioral factors males’ higher consumption of red meat, processed meats, and bread versus females’ preference for lower-fat foods, dairy, fruits, and vegetables and biological mechanisms. Notably, endogenous estrogen in females may confer protective effects against CRC development, while oral contraceptive use could further reduce female CRC risk (55).

Age-period-cohort model analyses revealed significant age-related increases in CRC burden, manifested by elevated age-standardized mortality rates (ASMR) and disability-adjusted life-years (DALY) rates peaking between 50 and 85 years, with gradual declines after age 70. Notably, males demonstrated disproportionately higher risks compared to females, particularly after age 50. While the exact mechanisms driving CRC burden escalation in younger adults (< 50 years) remain unclear, cohort effects suggest increased exposure to modifiable risk factors among those born in the latter half of the 20th century – including unhealthy diets, obesity, sedentary lifestyles, and rising smoking rates during early adulthood (56, 57). These findings underscore the urgency of prioritizing CRC screening and dietary interventions for elderly populations, alongside targeted health education campaigns emphasizing whole-grain consumption for males (58). The period effect showed a decreasing trend in disease burden, which may be attributed to increased survival due to the popularization of colonoscopy screening, increased intake of whole grain diet, and targeted therapeutic applications (e.g., anti-EGFR drugs) (59). The cohort effect also demonstrated a significantly lower burden of disease in later birth cohorts such as after 1980 than in earlier cohorts such as those born before 1940, possibly due to increased health awareness in the younger generation and increased intake of whole grains such as brown rice in place of refined rice in the daily diet, which reduces the risk of CRC.

In this study, a Bayesian age-period-cohort (BAPC) model was used to integrate the global burden of disease (GBD) data from 1990 to 2021 to predict the trend of the evolution of the burden of disease for low-grain diet-associated colorectal cancer (CRC) in 2022–2050, and the results showed that the global age-standardized mortality rate (ASMR) and the DALY rate (ASDR) in 2050 will respectively, decline to 3.086/100,000 and 69.10/100,000, which may be mainly attributed to screening universalization such as colonoscopy and dietary interventions such as WHO whole grain guideline promotion (60, 61). Despite the decline in standardization rates, attributable to the intensification of the social structure of the aging population, the absolute number of deaths and DALYs continues to rise, more prominently in the older population.

This study has some limitations, starting with the source of the data: the estimation of the GBD data relies on modeling assumptions that may underestimate the true impact of Diet low in whole grains, especially in rural areas or countries lacking dietary surveys. Secondly the interaction of other dietary factors such as red meat and alcohol with a low grain diet was not fully controlled for and may overestimate their independent effects. In addition, Diet low in whole grains thresholds are based on existing epidemiological studies, but individual metabolic differences may influence the actual risk. In future studies, it may be possible to conduct whole grain dose-effect studies to quantify the effects of whole grain intake on individual.

## 5 Conclusion

Age-standardized mortality rates and ASDR for CRC due to low whole grain diets declined globally between 1990 and 2021, but the absolute number of cases continues to increase. Low whole grain diet is an important modifiable factor in the disease burden of CRC, with significant age, sex, and regional heterogeneity in its impact. Bayesian BAPC model predictions showed a decreasing trend in ASMR and ASDR for colorectal cancer disease burden associated with low grain diets, but the absolute burden continued to increase due to increased aging. Decomposition analyses showed that population growth was the main cause of the increasing burden.

## Data Availability

The datasets presented in this study can be found in online repositories. The names of the repository/repositories and accession number(s) can be found below: https://vizhub.healthdata.org/gbd-results/.

## Footnotes

1
https://vizhub.healthdata.org/gbd-results/
