# Supplementary material for: Global burden of disease for colorectal cancer due to diet low in whole grains from 1990 to 2021 and its projections to 2050: analysis of the global burden of disease study 2021
Source: Front Nutr. 2025 Aug 7;12:1592425. doi: 10.3389/fnut.2025.1592425 (PMC12369419; doi:10.3389/fnut.2025.1592425)
Supplement: Supplementary file 1 [file Data_Sheet_1.pdf]

Global Burden of Disease for Colorectal Cancer due to Diet low in whole grains from 1990 to 2021 and Its Projections to 2050: Analysis of the Global Burden of Disease Study 2021

Supplementary material, including supplementary tables 1, 2, 3, 4 and supplementary figures 1, 2

Characteristics	Deaths		ASMR		EAPC
	1990（95%UI）	2021（95%UI）	1990（95%UI）	2021（95%UI）	
Global	101812.86(42588.20,151170.08)	186256.80(76126.73,284803.37)	2.79(1.17,4.15)	2.21(0.91,3.38)	-0.82
**Sex**					
Female	50191.26(20942.11,74789.98)	81912.42(34184.22,123364.04)	2.47(1.04,3.69)	1.76(0.74,2.66)	
Male	51621.60(21646.09,76727.84)	104344.38(42109.73,159294.07)	3.19(1.35,4.78)	2.75(1.11,4.20)	
**21GBD Regions**					
East Asia	22088.23(8770.80,34016.10)	52282.82(20975.52,83298.33)	2.78(1.10,4.27)	2.50(1.00,3.97)	-0.42
Southeast Asia	3950.46(1640.02,6042.76)	12528.26(5042.44,18992.75)	1.62(0.68,2.49)	2.01(0.81,3.04)	0.67
Oceania	30.40(12.27,47.51)	70.29(27.96,106.91)	1.17(0.47,1.81)	1.03(0.41,1.56)	-0.36
Central Asia	907.98(379.45,1365.31)	1163.26(480.67,1783.85)	1.95(0.82,2.94)	1.49(0.62,2.29)	-0.48
Central Europe	5914.65(2477.60,8741.12)	9495.24(3942.10,14239.80)	4.06(1.70,6.00)	4.13(1.71,6.20)	-0.05
Eastern Europe	9452.69(3919.93,14005.83)	12006.84(5039.47,17765.90)	3.41(1.41,5.04)	3.36(1.41,4.98)	-0.24
High-income Asia Pacific	1009.37(421.25,1533.18)	1479.30(601.99,2258.81)	4.35(1.82,6.61)	2.61(1.08,4.00)	-0.52
Australasia	5843.15(2460.66,8830.50)	13974.22(5930.76,21445.21)	3.03(1.28,4.59)	2.60(1.09,3.96)	-1.84
Western Europe	24784.51(10511.13,36960.19)	28984.77(11875.46,43653.13)	4.18(1.77,6.23)	2.79(1.16,4.19)	-1.34
Southern Latin America	1697.05(710.22,2524.85)	3019.80(1248.03,4597.84)	3.81(1.60,5.67)	3.39(1.40,5.17)	-0.14
Caribbean	13194.37(5633.05,19616.74)	15389.81(6563.53,23033.01)	3.67(1.57,5.45)	2.32(0.99,3.46)	0.20
High-income North America	636.70(271.92,959.90)	1426.41(562.20,2167.07)	2.57(1.09,3.86)	2.64(1.04,4.00)	-1.61
Central Latin America	310.96(132.58,475.22)	1024.08(391.53,1604.68)	1.61(0.69,2.46)	1.77(0.68,2.78)	0.84
Andean Latin America	925.82(380.44,1365.59)	3795.31(1513.46,5782.64)	1.20(0.49,1.77)	1.54(0.61,2.34)	0.37
Tropical Latin America	2637.90(1069.56,3947.36)	7009.92(2866.51,10716.52)	1.69(0.69,2.53)	1.68(0.69,2.57)	0.53
North Africa and Middle East	1426.88(590.48,2142.98)	5029.23(2143.76,7573.20)	1.69(0.70,2.55)	1.98(0.84,2.98)	0.19

South Asia	4280.28(1859.37,6563.94)	11355.69(4735.65,17076.82)	0.76(0.33,1.17)	0.79(0.33,1.18)	0.006
Central Sub-Saharan Africa	255.98(108.68,396.93)	645.35(261.03,1088.35)	1.95(0.82,3.04)	1.89(0.82,2.82)	0.10
Eastern Sub-Saharan Africa	1364.60(574.84,2131.20)	2773.89(1188.77,4139.98)	1.28(0.55,1.99)	1.31(0.54,2.23)	-0.24
Southern Sub-Saharan Africa	347.48(146.05,542.59)	969.29(408.64,1475.94)	1.38(0.58,2.15)	1.81(0.76,2.74)	1.00
Western Sub-Saharan Africa	753.40(307.40,1151.63)	1833.04(788.27,2756.76)	0.95(0.39,1.45)	1.07(0.46,1.60)	0.55
5 SDI Regions					
High-middle SDI	31689.75(13145.57,46872.58)	56721.44(22911.24,86094.51)	1.29(0.54,2.02)	1.23(0.51,1.84)	-0.55
High SDI	43042.62(18344.92,64228.50)	60473.36(25199.86,92184.17)	3.36(1.40,4.98)	2.89(1.17,4.38)	-1.24
Low-middle SDI	5747.62(2416.57,8914.23)	15442.23(6372.49,22973.55)	3.87(1.65,5.77)	2.70(1.12,4.10)	0.45
Low SDI	2751.01(1161.20,4291.23)	5639.91(2327.41,8439.87)	0.98(0.41,1.52)	1.11(0.46,1.65)	-0.24
Middle SDI	18434.85(7398.07,28070.12)	47737.84(19196.51,73112.02)	1.89(0.77,2.87)	1.85(0.74,2.83)	-0.13

TableS1:Changes in deaths, ASMR, and EAPC for ASMR, globally and in 21 super-regions and 5 SDI regions, 1990-2021

Characteristics	DALYS		ASDR		EAPC
	1990（95%UI）	2021（95%UI）	1990（95%UI）	2021（95%UI）	
Global	2540867.41(1050794.36,3754415.59)	4327218.86(1754865.24,6578232.30)	63.47(26.35,93.84)	50.19(20.37,76.30)	-0.83
Sex					
Female	1191104.02(486864.83,1765198.53)	1799227.21(736335.49,2717497.17)	55.85(22.91,82.83)	39.36(16.10,59.45)	
Male	1349763.39(563929.53,2011922.66)	2527991.65(1020943.86,3876411.05)	72.49(30.36,107.77)	62.39(25.20,95.64)	
21GBD Regions					
East Asia	646779.33(257105.92,994559.64)	1295744.16(524659.79,2051943.02)	69.28(27.54,106.65)	82.28(32.68,134.25)	-0.53
Southeast Asia	115594.06(47680.43,177859.55)	340389.08(136101.87,511101.24)	41.08(17.03,63.08)	61.99(25.07,94.67)	0.55
Oceania	932.53(373.02,1454.80)	2114.41(839.50,3230.69)	28.41(11.49,44.39)	23.55(9.19,35.48)	-0.38
Central Asia	25986.97(10919.29,39252.40)	31919.29(13173.55,48964.30)	52.45(22.00,79.19)	44.72(18.48,68.39)	-0.85
Central Europe	141071.81(58975.30,208941.72)	199028.25(82274.51,297981.99)	94.12(39.32,139.42)	127.75(52.80,192.67)	-0.15
Eastern Europe	240974.85(99959.43,357627.47)	273010.20(114998.80,401920.89)	85.55(35.48,126.94)	100.83(41.95,150.29)	-0.52
High-income Asia Pacific	142754.13(59701.38,214811.20)	250020.51(105027.07,380217.91)	70.50(29.53,106.10)	74.01(31.53,110.80)	-0.70
Australasia	23316.95(9745.30,35119.34)	29863.31(12591.41,45527.16)	100.80(42.14,151.66)	70.45(29.95,105.60)	-1.99
Western Europe	510577.72(215359.73,765412.67)	538523.35(222514.13,804551.11)	90.32(38.19,135.28)	76.06(31.44,112.36)	-1.34
Southern Latin America	38792.70(16122.62,57863.12)	65166.72(26905.01,99425.45)	83.94(34.95,125.16)	93.07(38.30,143.60)	-0.09
Caribbean	15190.28(6477.25,22949.97)	32388.67(12893.59,49039.06)	57.97(24.72,87.64)	62.76(25.11,96.34)	0.26
High-income North America	286863.11(121592.40,423917.97)	342012.82(144493.66,508975.16)	83.39(35.33,123.23)	68.33(28.85,101.93)	-1.33
Central Latin America	24186.86(9945.87,35390.33)	97675.63(39088.78,148606.43)	27.34(11.24,40.18)	42.83(17.26,65.32)	-0.09
Andean Latin America	7809.32(3322.01,11899.94)	23978.96(9183.44,37638.08)	36.67(15.64,55.90)	39.51(15.26,61.51)	0.29
Tropical Latin America	38033.19(15692.38,56901.27)	126985.67(54321.21,191290.85)	39.42(16.28,59.13)	54.37(23.18,82.73)	0.64
North Africa and Middle East	75303.21(31144.43,112922.27)	188025.27(75807.81,285507.19)	41.27(16.87,62.03)	43.03(17.78,65.69)	-0.02

South Asia	131068.08(56844.82,200092.07)	320917.87(133326.71,481047.01)	20.12(8.75,30.74)	21.35(8.60,31.86)	-0.09
Central Sub-Saharan Africa	7453.95(3137.57,11526.74)	18960.89(7606.00,31746.56)	30.78(13.03,47.79)	36.79(14.76,61.19)	0.06
Eastern Sub-Saharan Africa	38963.53(16325.85,60926.79)	75702.65(31963.04,112168.64)	48.22(20.27,75.34)	53.07(21.99,79.50)	-0.62
Southern Sub-Saharan Africa	9459.54(3978.87,14869.29)	26263.05(11059.34,39903.23)	32.51(13.66,50.92)	52.28(22.41,79.43)	1.10
Western Sub-Saharan Africa	19755.27(8019.21,30293.29)	48528.11(20548.63,73775.70)	21.74(8.89,33.34)	25.97(11.32,38.86)	0.39
5 SDI Regions					
High-middle SDI	808497.77(332628.85,1194206.54)	1298819.51(519591.61,1965735.77)	80.20(33.06,118.50)	66.60(26.65,100.77)	-0.70
High SDI	941428.05(398133.00,1402353.53)	1205653.28(500387.21,1815640.18)	86.30(36.51,128.53)	60.69(25.12,91.16)	-1.20
Low-middle SDI	170153.86(70941.07,262875.67)	430073.38(176445.15,643409.64)	25.28(10.62,39.13)	28.05(11.53,41.95)	0.36
Low SDI	79389.16(33441.34,123672.58)	157203.40(64961.68,233832.04)	32.35(13.68,50.39)	28.75(11.87,42.88)	-0.51
Middle SDI	537903.75(215133.74,819572.94)	1230181.64(494733.14,1879458.67)	47.61(19.10,72.49)	44.72(17.98,68.32)	-0.25

TableS2:Changes in DALYs, ASDRs, and EAPCs for ASDRs, globally and in 21 super-regions and 5 SDI regions, 1990-2021

	age	val	low	up	type	sex_name	location_name	location_id
1	27.5	10.58081227759399	10.24500589439036	10.92762557754933	Age effect	Both	Global	1
2	32.5	19.86544988871997	19.38363110904153	20.35924523435475	Age effect	Both	Global	1
3	37.5	31.12353835273475	30.49773447091409	31.76218353261561	Age effect	Both	Global	1
4	42.5	47.08894804801308	46.28033061582754	47.91169377493924	Age effect	Both	Global	1
5	47.5	73.33086053652534	72.24032756585436	74.43785608703492	Age effect	Both	Global	1
6	52.5	110.7133130576129	109.3288319964165	112.1153264364406	Age effect	Both	Global	1
7	57.5	152.8879528924051	151.1594860958298	154.6361842273764	Age effect	Both	Global	1
8	62.5	196.9917483114797	194.908499867637	199.0972632243666	Age effect	Both	Global	1
9	67.5	238.5612307452509	236.0882679991857	241.0600971281011	Age effect	Both	Global	1
10	72.5	279.9439431065707	276.9599516370474	282.9600843690066	Age effect	Both	Global	1
11	77.5	313.0461837872959	309.442740603094	316.6915888632406	Age effect	Both	Global	1
12	82.5	336.5562091728253	331.7167351853299	341.4662871003437	Age effect	Both	Global	1
13	87.5	368.8440922465375	362.26486328832	375.5428090659033	Age effect	Both	Global	1

			31					
14	92.5	426.3487941132928	415.6940177857751	437.2766661644249	Age effect	Both	Global	1
15	97.5	451.9377612357459	432.9338410109659	471.7758712366507	Age effect	Both	Global	1
16	1897	1.334622810605737	1.155368652629103	1.541688051286403	Cohort RR	Both	Global	1
17	1902	1.298594348789619	1.218758157947159	1.383660303491851	Cohort RR	Both	Global	1
18	1907	1.279151563858576	1.233784489355173	1.326186815800385	Cohort RR	Both	Global	1
19	1912	1.287139833545973	1.256069383646209	1.318978849951333	Cohort RR	Both	Global	1
20	1917	1.202922894038608	1.179989828857623	1.226301662619514	Cohort RR	Both	Global	1
21	1922	1.222904642294168	1.20448491894076	1.241606051373217	Cohort RR	Both	Global	1
22	1927	1.188071420973225	1.17265033717121	1.203695301651759	Cohort RR	Both	Global	1
23	1932	1.126751705670127	1.113300778885961	1.140365146875178	Cohort RR	Both	Global	1
24	1937	1.065692309174482	1.053610051897617	1.077913119553267	Cohort RR	Both	Global	1
25	1942	1	1	1	Cohort RR	Both	Global	1
26	1947	0.9540973179969341	0.9432240668384159	0.9650959132755961	Cohort RR	Both	Global	1

27	1952	0.928559487369024	0.9175797362236803	0.9396706221211046	Cohort RR	Both	Global	1
28	1957	0.8824844113795749	0.871067043172957	0.8940514308648024	Cohort RR	Both	Global	1
29	1962	0.8313129509285115	0.8192383352798629	0.8435655323006669	Cohort RR	Both	Global	1
30	1967	0.8070088905956958	0.7936051802377602	0.8206389848732848	Cohort RR	Both	Global	1
31	1972	0.7676355344003871	0.7523732639352104	0.7832074077062255	Cohort RR	Both	Global	1
32	1977	0.7198823435929516	0.702243983393525	0.7379637289487082	Cohort RR	Both	Global	1
33	1982	0.6869029020688927	0.6658978680888753	0.7085705173149652	Cohort RR	Both	Global	1
34	1987	0.6808990360347301	0.6537428922250158	0.7091832321038029	Cohort RR	Both	Global	1
35	1992	0.6303680104349144	0.589319590467007	0.6742756137884046	Cohort RR	Both	Global	1
36	1994.5	1.091350190926447	1.080577257837477	1.102230525949428	Period RR	Both	Global	1
37	1999.5	1.039544591963912	1.02962543258955	1.049559310091563	Period RR	Both	Global	1
38	2004.5	1	1	1	Period RR	Both	Global	1
39	2009.5	0.9417466538292573	0.9330841670240984	0.9504895606868632	Period RR	Both	Global	1
40	2014.5	0.9033068769466215	0.8949852230442	0.9117059063428642	Period RR	Both	Global	1

			834					
41	2019.5	0.8899894096431695	0.8817065524698227	0.8983500769708834	Period RR	Both	Global	1
42	27.5	-0.933757363	-1.141524545	-0.725553524	Local Drifts	Both	Global	1
43	32.5	-0.879319119	-1.009937879	-0.748528005	Local Drifts	Both	Global	1
44	37.5	-0.986256344	-1.086940426	-0.885469775	Local Drifts	Both	Global	1
45	42.5	-0.978429435	-1.059618839	-0.897173407	Local Drifts	Both	Global	1
46	47.5	-0.891933997	-0.959302725	-0.824519443	Local Drifts	Both	Global	1
47	52.5	-0.874029028	-0.932554124	-0.815469358	Local Drifts	Both	Global	1
48	57.5	-0.935053337	-0.988027931	-0.8820504	Local Drifts	Both	Global	1
49	62.5	-0.956533709	-1.006337558	-0.906704803	Local Drifts	Both	Global	1
50	67.5	-1.020398808	-1.06975362	-0.971019373	Local Drifts	Both	Global	1
51	72.5	-1.031104827	-1.084330879	-0.977850135	Local Drifts	Both	Global	1
52	77.5	-0.79089972	-0.854349582	-0.727409252	Local Drifts	Both	Global	1
53	82.5	-0.665849633	-0.744279361	-0.587357931	Local Drifts	Both	Global	1
54	87.5	-0.489136821	-0.602019498	-0.376125948	Local Drifts	Both	Global	1
55	92.5	-0.369218417	-0.563315595	-0.174742367	Local Drifts	Both	Global	1
56	97.5	-0.376693182	-0.805458759	0.053925723301	Local Drifts	Both	Global	1
57	1	-0.854189605	-0.890229391	-0.818136713	Net drift	Both	Global	1

TableS3: Results of the analysis of the APC model of mortality

	age	val	low	up	type	sex_name	location_name	location_id
1	27.5	10.58081227759399	10.24500589439036	10.92762557754933	Age effect	Both	Global	1
2	32.5	19.86544988871997	19.38363110904153	20.35924523435475	Age effect	Both	Global	1
3	37.5	31.12353835273475	30.49773447091409	31.76218353261561	Age effect	Both	Global	1
4	42.5	47.08894804801308	46.28033061582754	47.91169377493924	Age effect	Both	Global	1
5	47.5	73.33086053652534	72.24032756585436	74.43785608703492	Age effect	Both	Global	1
6	52.5	110.7133130576129	109.3288319964165	112.1153264364406	Age effect	Both	Global	1
7	57.5	152.8879528924051	151.1594860958298	154.6361842273764	Age effect	Both	Global	1
8	62.5	196.9917483114797	194.908499867637	199.0972632243666	Age effect	Both	Global	1
9	67.5	238.5612307452509	236.0882679991857	241.0600971281011	Age effect	Both	Global	1
10	72.5	279.9439431065707	276.9599516370474	282.9600843690066	Age effect	Both	Global	1
11	77.5	313.0461837872959	309.442740603094	316.6915888632406	Age effect	Both	Global	1
12	82.5	336.5562091728253	331.7167351853299	341.4662871003437	Age effect	Both	Global	1
13	87.5	368.8440922465375	362.2648632883231	375.5428090659033	Age effect	Both	Global	1
14	92.5	426.3487941132928	415.694017785775	437.2766661644249	Age effect	Both	Global	1

			1					
15	97.5	451.9377612357459	432.9338410109659	471.7758712366507	Age effect	Both	Global	1
16	1897	1.334622810605737	1.155368652629103	1.541688051286403	Cohort RR	Both	Global	1
17	1902	1.298594348789619	1.218758157947159	1.383660303491851	Cohort RR	Both	Global	1
18	1907	1.279151563858576	1.233784489355173	1.326186815800385	Cohort RR	Both	Global	1
19	1912	1.287139833545973	1.256069383646209	1.318978849951333	Cohort RR	Both	Global	1
20	1917	1.202922894038608	1.179989828857623	1.226301662619514	Cohort RR	Both	Global	1
21	1922	1.222904642294168	1.20448491894076	1.241606051373217	Cohort RR	Both	Global	1
22	1927	1.188071420973225	1.17265033717121	1.203695301651759	Cohort RR	Both	Global	1
23	1932	1.126751705670127	1.113300778885961	1.140365146875178	Cohort RR	Both	Global	1
24	1937	1.065692309174482	1.053610051897617	1.077913119553267	Cohort RR	Both	Global	1
25	1942	1	1	1	Cohort RR	Both	Global	1
26	1947	0.9540973179969341	0.9432240668384159	0.9650959132755961	Cohort RR	Both	Global	1
27	1952	0.928559487369024	0.9175797362236803	0.9396706221211046	Cohort RR	Both	Global	1
28	1957	0.8824844113795749	0.871067043172957	0.8940514308648024	Cohort RR	Both	Global	1

29	1962	0.8313129509285115	0.8192383352798629	0.8435655323006669	Cohort RR	Both	Global	1
30	1967	0.8070088905956958	0.7936051802377602	0.8206389848732848	Cohort RR	Both	Global	1
31	1972	0.7676355344003871	0.7523732639352104	0.7832074077062255	Cohort RR	Both	Global	1
32	1977	0.7198823435929516	0.702243983393525	0.7379637289487082	Cohort RR	Both	Global	1
33	1982	0.6869029020688927	0.6658978680888753	0.7085705173149652	Cohort RR	Both	Global	1
34	1987	0.6808990360347301	0.6537428922250158	0.7091832321038029	Cohort RR	Both	Global	1
35	1992	0.6303680104349144	0.589319590467007	0.6742756137884046	Cohort RR	Both	Global	1
36	1994.5	1.091350190926447	1.080577257837477	1.102230525949428	Period RR	Both	Global	1
37	1999.5	1.039544591963912	1.02962543258955	1.049559310091563	Period RR	Both	Global	1
38	2004.5	1	1	1	Period RR	Both	Global	1
39	2009.5	0.9417466538292573	0.9330841670240984	0.9504895606868632	Period RR	Both	Global	1
40	2014.5	0.9033068769466215	0.8949852230442834	0.9117059063428642	Period RR	Both	Global	1
41	2019.5	0.8899894096431695	0.8817065524698227	0.8983500769708834	Period RR	Both	Global	1
42	27.5	-0.933757363	-1.141524545	-0.725553524	Local Drifts	Both	Global	1
43	32.5	-0.879319119	-1.009937879	-0.748528005	Local Drifts	Both	Global	1

44	37.5	-0.986256344	-1.086940426	-0.885469775	Local Drifts	Both	Global	1
45	42.5	-0.978429435	-1.059618839	-0.897173407	Local Drifts	Both	Global	1
46	47.5	-0.891933997	-0.959302725	-0.824519443	Local Drifts	Both	Global	1
47	52.5	-0.874029028	-0.932554124	-0.815469358	Local Drifts	Both	Global	1
48	57.5	-0.935053337	-0.988027931	-0.8820504	Local Drifts	Both	Global	1
49	62.5	-0.956533709	-1.006337558	-0.906704803	Local Drifts	Both	Global	1
50	67.5	-1.020398808	-1.06975362	-0.971019373	Local Drifts	Both	Global	1
51	72.5	-1.031104827	-1.084330879	-0.977850135	Local Drifts	Both	Global	1
52	77.5	-0.79089972	-0.854349582	-0.727409252	Local Drifts	Both	Global	1
53	82.5	-0.665849633	-0.744279361	-0.587357931	Local Drifts	Both	Global	1
54	87.5	-0.489136821	-0.602019498	-0.376125948	Local Drifts	Both	Global	1
55	92.5	-0.369218417	-0.563315595	-0.174742367	Local Drifts	Both	Global	1
56	97.5	-0.376693182	-0.805458759	0.05392572330189527	Local Drifts	Both	Global	1
57	1	-0.854189605	-0.890229391	-0.818136713	Net drift	Both	Global	1

TableS4: Results of the analysis of the APC model for the rate of DALYs

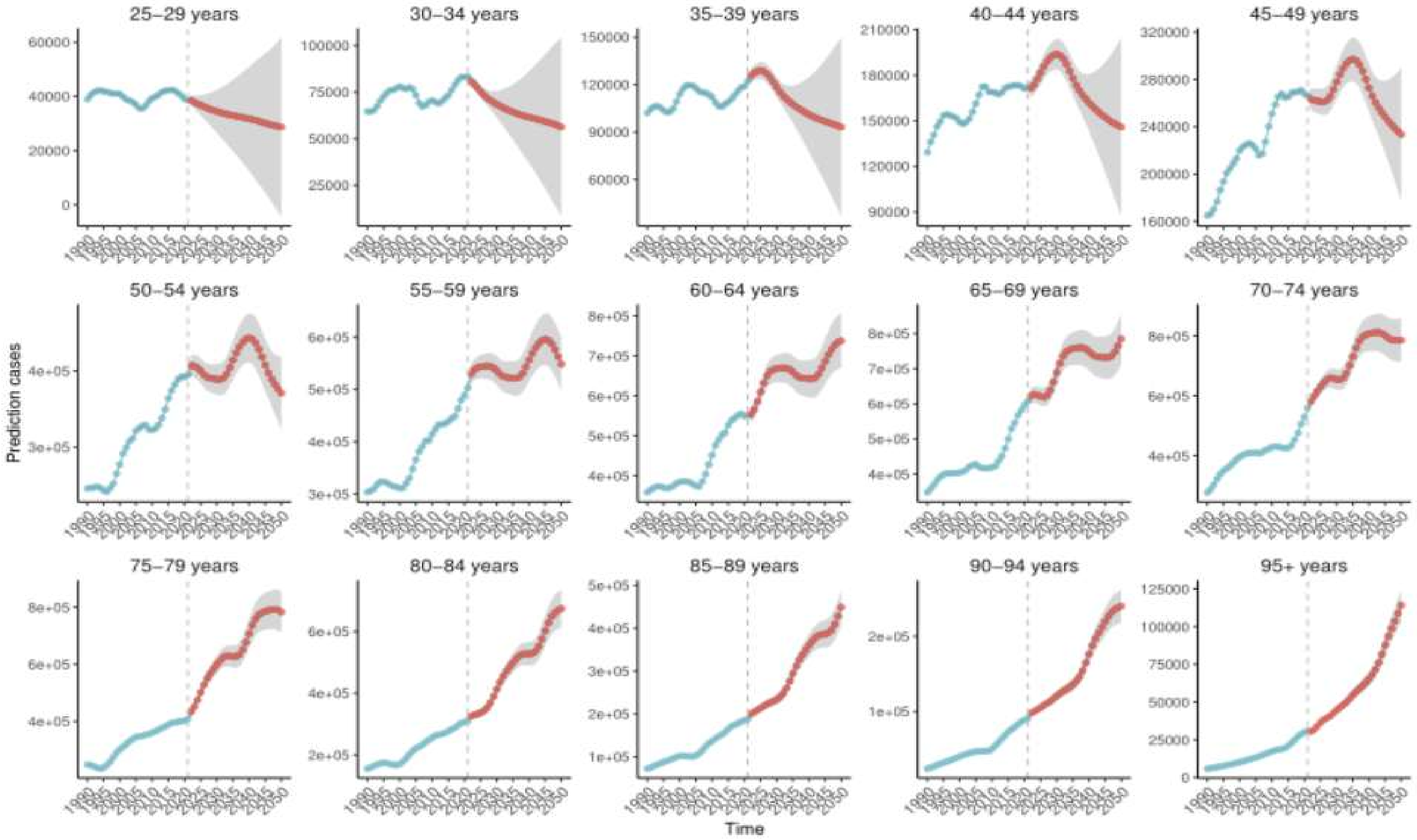

Supplementary Figure 1：DALYs by age as predicted by the BAPC model in 2050

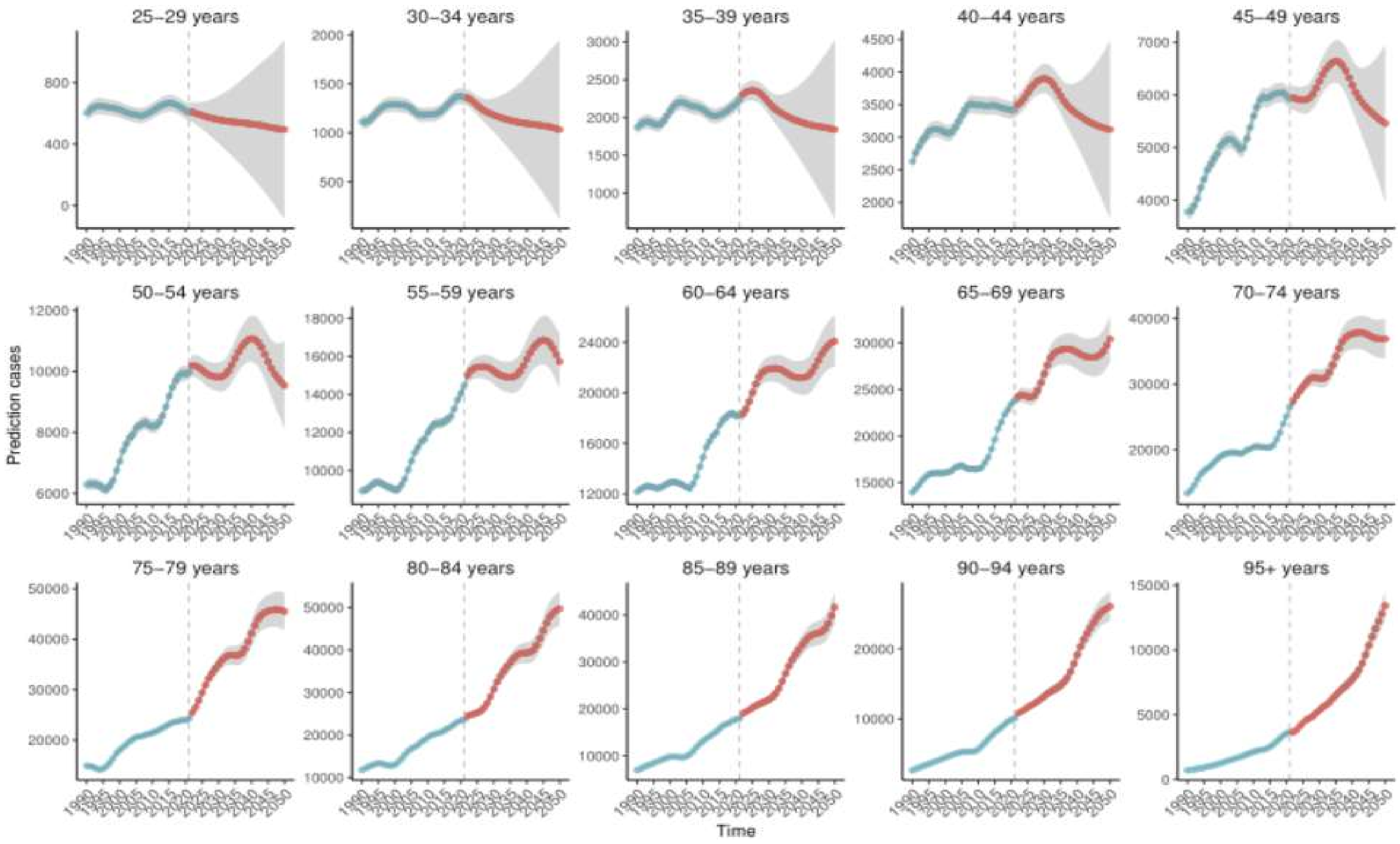

Supplementary Figure 2：Deaths at different ages predicted by the BAPC model in 2050
